# Thaumarchaeal ammonium oxidation and evidence for a nitrogen cycle in a subsurface radioactive thermal spring in the Austrian Central Alps

**DOI:** 10.3389/fmicb.2014.00225

**Published:** 2014-05-16

**Authors:** Friedrich W. Gerbl, Gerhard W. Weidler, Wolfgang Wanek, Angelika Erhardt, Helga Stan-Lotter

**Affiliations:** ^1^Division of Molecular Biology, University of SalzburgSalzburg, Austria; ^2^Bioanalyticum, Institut für Mikrobiologie und Hygiene, Dr. Reisinger e. U.Perg, Austria; ^3^Department of Microbiology and Ecosystem Science, University of ViennaVienna, Austria; ^4^Analytec, Labor für Lebensmitteluntersuchung und UmweltanalytikSalzburg, Austria

**Keywords:** subsurface thermal spring, *Thaumarchaeota*, archaeal ammonia oxidation, nitrogen cycle, functional genes, stable isotope probing

## Abstract

Previous studies had suggested the presence of ammonium oxidizing *Thaumarchaeota* as well as nitrite oxidizing *Bacteria* in the subsurface spring called Franz Josef Quelle (FJQ), a slightly radioactive thermal mineral spring with a temperature of 43.6–47°C near the alpine village of Bad Gastein, Austria. The microbiological consortium of the FJQ was investigated for its utilization of nitrogen compounds and the putative presence of a subsurface nitrogen cycle. Microcosm experiments made with samples from the spring water, containing planktonic microorganisms, or from biofilms, were used in this study. Three slightly different media, enriched with vitamins and trace elements, and two incubation temperatures (30 and 40°C, respectively) were employed. Under aerobic conditions, high rates of conversion of ammonium to nitrite, as well as nitrite to nitrate were measured. Under oxygen-limited conditions nitrate was converted to gaseous compounds. Stable isotope probing with ^15^NH_4_Cl or (^15^NH_4_)_2_SO_4_as sole energy sources revealed incorporation of ^15^N into community DNA. Genomic DNA as well as RNA were extracted from all microcosms. The following genes or fragments of genes were successfully amplified, cloned and sequenced by standard PCR from DNA extracts: Ammonia monooxygenase subunit A (*amoA*), nitrite oxidoreductase subunits A and B (*nxrA* and *nxrB*), nitrate reductase (*narG*), nitrite reductase (*nirS*), nitric oxide reductases (*cnorB* and *qnorB*), nitrous oxide reductase (*nosZ*). Reverse transcription of extracted total RNA and real-time PCR suggested the expression of each of those genes. Nitrogen fixation (as probed with *nifH* and *nifD*) was not detected. However, a geological origin of NH^+^_4_ in the water of the FJQ cannot be excluded, considering the silicate, granite and gneiss containing environment. The data suggested the operation of a nitrogen cycle in the subsurface environment of the FJQ.

## Introduction

The knowledge of microbiological activities in the global nitrogen cycle was significantly extended in the last few years. Many studies provided convincing data in support of archaea controlling the fate of ammonia in terrestrial, marine, and geothermal habitats (see Stahl and de la Torre, 2012, for a recent review). Ammonia-oxidizing archaea (AOA) are now thought to be an important ammonia-oxidizing population in natural environments. Although their contribution to soil nitrification is still under debate, recent reports were able to distinguish between the activities of ammonia-oxidizing bacteria (AOB) and AOA (Eloy Alves et al., 2013; Taylor et al., 2013). In the oceans, ammonia concentrations are extremely low (below 5 nM; Agogué et al., 2008), which led to the assumption that ammonia as an energy source is rather unlikely. However, reports of a very high affinity of AOAs to ammonia (Martens-Habbena et al., 2009) suggested that oligotrophic AOAs are equipped to compete for ammonium as energy source in nutrient-deprived waters.

The microbiological exploration of subsurface environments has increased greatly, because the highest numbers and diversity of microorganisms are found within subterranean and submarine environments as well as in the oceans (Whitman et al., 1998). With increasing discoveries of microbial communities in the deep subsurface, the problem of availability of nutrients has come into focus (Silver et al., 2012). Nitrogen in subsurface ecosystems could be NH_3_ or NH^+^_4_, originating either from organic substrates, if present, or from N_2_ or NH^+^_4_ containing fluids in silicates and gas reservoirs (Holloway and Dahlgren, 2002; Silver et al., 2012).

Bad Gastein, a village in the Alps near Salzburg, Austria, is known for its thermal mineral springs. The open joint system of the springs enables the infiltration of surface water to depths of up to 3000 m (Lettner et al., 1996). There the water becomes heated and loaded with radon and trace elements. The waters are of meteoric origin of 3600–3800 years of age (Lettner et al., 1996). Seventeen thermal springs are delivering 4.5 million liters of water per day, with temperatures between 35 and 47°C (Lettner et al., 1996). One of the major springs is the Franz Josef Quelle (FJQ). It is located in a gallery which was driven horizontally more than 100 m into the rock of the steep flank of the Gastein mountain (Zötl, 1995). At the end of the dark gallery the thermal, slightly radioactive water pours out of the rock, delivering some 8–12,000 l/h (Zötl, 1995; Weidler et al., 2007). The main spring can be reached by a small window cut through the rock from above and covered with a removable metal-framed glass lid. Several secondary springs with a lower output originate from cracks and fissures in the vertical rock surface next to the main spring. Submerged pebbles and parts of the rock are heavily colonized by microorganisms, forming microbial mats (Weidler et al., 2007). The FJQ spring thus provides an easy access (although not for the general public) to the depth of a thermal subsurface environment. We published studies on the microbial communities of the FJQ spring, which showed that they consisted of archaea and bacteria (Weidler et al., 2007, 2008; Dornmayr-Pfaffenhuemer et al., 2011; Gerbl et al., 2012). Evidence was obtained that ammonium oxidation in the spring is carried out by certain mesophiles within the phylum *Crenarchaeota* (Weidler et al., 2007, 2008). Recently the archaeal lineage to which AOA affiliate has been re-named *Thaumarchaeota* (Brochier-Armanet et al., 2008). The energy source for this and similar subterranean microbial communities might possibly be ammonia. If this is correct, a putative nitrogen cycle might be operative in the FJQ. The concentrations of ammonia in many natural environments are generally very low, which is also valid for the FJQ. It is therefore especially worthwhile to investigate this oligotrophic environment for the contribution of archaea and bacteria to a presumed nitrogen cycle. The microbial abilities for using ammonium compounds as energy sources in the FJQ spring were investigated in this study. An analysis of the transformation of nitrogen compounds and the presence of the required genetic inventory was performed.

## Materials and methods

### Sampling site, chemical analysis, and sample collection

Water samples for planktonic microorganisms were taken from three water discharges which emerged from rock fissures into a small basin (see Weidler et al., 2007). The water temperature in the basin was 43.6°C at a pH of about 8. *In situ* measurements of the O_2_ content of the water within the basin revealed up to 1.9 mg L^−1^ dissolved oxygen in the thermal spring water. Chemical analyses of the spring water were performed by a commercial laboratory (Hydrologische Untersuchungsstelle, Salzburg, Austria). Microbial mats (biofilm material) were taken from the surrounding of a very small water efflux near the bottom of the wall of the gallery (see Weidler et al., 2007). A sterilized 5 L glass bottle was used for sampling the thermal mineral water under aseptic conditions. This water was subsequently decanted into a sterile 25 L plastic receptacle. In sum, 75 L of thermal mineral spring water were collected at each sampling trip. Biofilm material was collected using a sterile spoon and sterile 50 mL reaction tubes. Samples were brought to the laboratory within 2 h after sampling for further processing.

### Aerobic microcosm experiments

Microorganisms were concentrated by filtering the spring water through a Stericup® Filter unit with a pore size of 0.22 μm (Millipore, MA, USA). Filters containing the solid (planktonic part) of about 25 L of thermal spring water were excised with autoclaved tweezers and scalpel and immediately used as inocula for microcosms. Alternatively, microcosms were inoculated with 35 mL of biofilm material. Three different media designated as Wuchter, Simon or FG medium, respectively, were used for the preparation of microcosms. Each medium was made up with 2.5 L of sterile filtered mineral spring water from the FJQ. Simon medium contained (in mM): KH_2_PO_4_, 5; Na_2_HPO_4_, 5; (NH_4_)_2_SO_4_, 1; KCl, 2 (Simon et al., 2005, except that the antibiotics of the original medium were omitted). Wuchter medium contained (in mM): NH_4_Cl, 0.5; NaNO_3_, 0.1; NaH_2_PO_4_, 0.1; NaHCO_3_, 0.1 (Wuchter et al., 2006). The third medium (FG medium) was developed for this study and contained (in mM): (NH_4_)_2_SO_4_, 2.5; NaCl, 1; CaCl_2_ × 2H_2_O, 1; NaHCO_3_, 1; KH_2_PO_4_, 1; MgSO_4_ × 7H_2_O, 1; CuSO_4_, 0.05. Finally, 1% (v/v) of a trace element solution and of a vitamin solution as described by Wolin et al. (1963) were added to each medium. Control experiments were performed using the ammonia-oxidizing chemolithoautotrophic bacterium *Nitrosomonas europaea* (a gift of Graeme Nicol, University of Aberdeen), which was cultivated using the mineral medium described by Schmidt et al. (2004). For microcosms which were prepared for stable isotope probing (SIP), ^15^N labeled nitrogen compounds (^15^NH_4_Cl, [^15^NH_4_]_2_SO_4_) were used.

### Stable isotope probing

Microbiological samples within microcosms for SIP were incubated for at least 8 weeks at 40°C, followed by extraction of DNA (see below). Ultracentrifugation of extracted DNA was carried out in polyallomer Ultracrimp® tubes (13.5 mL, Sorvall®) in a Sorvall® Discovery™ 100SE Ultracentrifuge (Kendro Laboratory Products, Asheville, USA), equipped with a STEP*SAVER*™ 50V39 vertical rotor containing cartridges for 13.5 mL Ultracrimp® tubes, in accordance to a protocol, published by Neufeld et al. (2007). Slight modifications due to the larger volume of the centrifugation tubes were as follows: 80–100 μg DNA were re-suspended in a volume of 1.0 mL of 1 × TE buffer (100 mM Tris-Cl, pH 7.6, 10 mM EDTA) and 1.0 g CsCl powder was added, followed by dissolving the CsCl by gentle shaking. Twelve milliliter of a CsCl solution (1 g mL^−1^ CsCl dissolved in 1 × TE buffer) were added to the DNA-CsCl sample and mixed gently. The mixture was then transferred to ultracentrifugation tubes and 100 μL of a 10 mg mL^−1^ ethidium bromide solution was added per tube. Tubes were sealed as recommended by the manufacturer and samples were centrifuged at 177,000 × g for 40 h at 20°C. DNA bands which formed during density gradient centrifugation were visualized under UV illumination (UV-Transilluminator Combi Light TFC-20 M, Vilber Lourmat, France), and images were taken with a digital camera (Nikon Coolpix E4500). DNA bands were isolated using a syringe and a needle. Extracted DNA was cleaned and precipitated as described by Neufeld et al. (2007). DNA concentrations were measured using a NanoDrop® ND-1000 spectrophotometer (Thermo Fisher Scientific Inc., Wilmington, USA). Distinct DNA bands obtained from two independent microcosms from different sampling trips and from each type of medium were chosen and a volume containing about 2.5 μg of DNA from each band was deep frozen. These samples were transferred to the Department of Microbiology and Ecosystem Science at the University of Vienna for measurement of the isotopic composition (at% ^15^N) by isotope ratio mass spectrometry (IRMS) after addition of standards as described recently (Dolinšek et al., 2013). From the DNA collected from the fractions of the density gradient, 50 μL each of DNA samples were pipetted into tin capsules and 7 μg nitrogen were added in the form of a glycine stock solution. This standard addition (spike) was necessary to enable measurements of the low N amounts present in the DNA samples (as low as 0.1 μg N), which had ^15^N enrichments of up to 99 at% ^15^N. After spiking the samples they were dried in a drying oven at 60°C overnight and analyzed by an elemental analyzer (EA 1110, CE Instruments) coupled via a ConFlo III device to the IRMS instrument (Delta Plus, Thermo Fisher). Similar aliquots of glycine stock solution were also analyzed for N content and isotope ratio without addition of DNA. The ^15^N enrichment of DNA was calculated as follows:

$$\begin{array}{l} \text{​​​​​​at}{\%}^{\text{15}}{\text{N}}_{\text{DNA}}\text{=}\\ \;(\text{at}\%{\;}^{\text{15}}{\text{N}}_{\text{total}}\times {\text{N}}_{\text{total}}-\text{at}{\%}^{\text{15}}{\text{N}}_{\text{gly}}\times {\text{N}}_{\text{gly}})\text{/}({\text{N}}_{\text{total}}-{\text{N}}_{\text{gly}}) \end{array}$$

where at% ^15^N represents the ^15^N enrichment of N (in %) and N the amount of N in each tin capsule (in μg N), and the subscripts DNA, total and gly represent the representative values for DNA, glycine + DNA and glycine, respectively, (Hannon and Böhlke, 2008; Dolinšek et al., 2013).

### Probing for functional genes

Microcosms designed for investigation of functional genes were first incubated for 8 weeks under aerobic conditions (see above). Then half of the medium and approximately half of the biological material were filtered through a filter unit. Following excision of filters, the biological material was subjected to extraction of RNA and DNA as described by Hurt et al. (2001), using the Qiagen® RNA-DNA Midi Kit (Qiagen, Hilden, Germany), with some modifications as follows: Four milliliter of denaturation buffer were added to the excised filter in a sterile mortar and the sample was frozen with liquid nitrogen and ground with an autoclaved pestle until thawed. Freezing and grinding were repeated three times. After final thawing, the samples were transferred to a 50 mL polypropylene reaction tube and 18 mL of extraction buffer (without SDS) were added. Subsequently 7.5 mL of 10% SDS (w/v) and 200 μL proteinase K (10 mg mL^−1^) were added, samples were intensely mixed and incubated for up to 1 h at 65°C, while inverting the tubes every 10 min. Separation and purification of RNA and DNA was carried out as recommended by the manufacturer.

The remaining medium of each microcosm, including the biological material, was passed through a Stericup® Filter Unit. The filters were excised and transferred to a sterile glass flask, containing 300 mL of primary medium (Wuchter, Simon or FG medium, respectively). One milliliter of 1 M NaNO_3_ was added to each microcosm resulting in a final concentration of about 250 mg L^−1^ NO^−^_3_. Flasks were placed into an anaerobic chamber and oxygen-limited conditions were created with Anaerocult® A bags (Merck Chemicals, Darmstadt, Germany). Incubation was carried out at 40°C. Anaerobic chambers were opened once a week to monitor consumption of nitrate. The amount of nitrate was adjusted to 250 mg L^−1^ if necessary. pH values were examined weekly and adjusted as well. After five additional weeks of incubation of the microcosms under oxygen-limited conditions, RNA and DNA were extracted as described above.

### Standard PCR of enzymes involved in nitrogen transformations

Several primer pairs were used for PCR amplification of genes of different phylogenetic groups of microorganisms and are displayed in Table 1. PCR reactions were carried out in a total volume of 50 μL with 1.25 U of DreamTag™ polymerase (Fermentas, Life Science Inc., USA) as recommended by the manufacturer. Fifty to one hundred and fifty nanogram DNA (depending on the template DNA and the primer pairs) and 50–100 pM of both forward and reverse primer were used. The general conditions for PCR are indicated in the references for the respective primers (see Table 1). Optimal annealing temperatures were determined by PCR reactions over a range of temperatures. For primers targeting *narG*, *cnorB*, and *qnorB*, amplification was done using a touchdown protocol as follows: for the first 10 cycles the annealing temperature was decreased by 0.5°C per cycle, starting from 60°C, followed by 25 cycles with an annealing temperature of 55°C (*narG*), or starting from 57°C, followed by 30 cycles with an annealing temperature of 55°C (*cnorB* and *qnorB*), respectively. PCR products were separated by gel electrophoresis and products of the expected size were excised from the gel, followed by recovery and purification using a QIAquick® Gel Extraction Kit (Qiagen, Hilden, Germany), as recommended by the manufacturer.

**Table 1:** **Primers targeting fragments of genes encoding nitrogen metabolizing enzymes**.

**Primer**	**Sequence 5′ to 3′**	**Target gene fragment**	**Fragment, length [bp]**	**References**
CrenAmo1F	AAT GGT CTG GCT WAG ACG C	Crenarchaeal *amoA*	640	Könneke et al., 2005
CrenAmo1R	GAC CAR GCG GCC ATC CA			
amoA19F	ATG GTC TGG CTW AGA CG	Archaeal *amoA*	624	Leininger et al., 2006
amo643R	TCC CAC TTW GAC CAR GCG GCC ATC CA			Treusch et al., 2005
Arch-amoAF	CTG AYT GGG CYT GGA CAT C	Archaeal *amoA*	256	Wuchter et al., 2006
Arch-amoAR	TTC TTC TTT GTT GCC CAG TA			
F151	TGG GAR CGT GTG TAT CAC G	*nxrA*	663	Koch, 2009
R831	CGT GCC AGG TRT AGT T			
nxrBF169	TAC ATG TGG TGG AAC A	All known *Nitrospira*-like *nxrB*	485	Maixner, 2009
nxrBR638	CGG TTC TGG TCR ATC A			
nxrBF19	TGG CAA CTG GGA CGG AAG ATG	*nxrB Nitrospira*	1239	Maixner, 2009
nxrBR1237	GTA GAT CGG CTC TTC GAC CTG			Lücker et al., 2010
narG1960F	TAY GTS GGS CAR GAR AA	*narG*	650	Philippot et al., 2002
narG2650R	TTY TCR TAC CAB GTB GC			
nirS1F	CCT AYT GGC CGC CRC ART	*nirS*	890	Braker et al., 1998
nirS6R	CGT TGA ACT TRC CGG T			
nirS1F	CCT AYT GGC CGC CRC ART	*nirS*	257	Braker et al., 1998
nirS3R	GCC GCC GTC RTG VAG GAA			
cnorB2F	GAC AAG NNN TAC TGG TGG T	*cnorB*	454	Braker and Tiedje, 2003
cnorB7R	TGN CCR TGN GCN GCN GT			
qnorB2F	GGN CAY CAR GGN TAY GA	*qnorB*	262	Braker and Tiedje, 2003
qnorB5R	ACC CAN AGR TGN ACN ACC CAC CA			
Nos661F	CGG CTG GGG GCT GAC CAA	*nosZ*	1150	Scala and Kerkhof, 1998
Nos1773R	ATR TCG ATC ARC TGB TCG TT			
amoA1F	GGG GTT TCT ACT GGT GGT	Bacterial *amoA*	491	Rotthauwe et al., 1997
amoA2R	CCC CTC KGS AAA GCC TTC TTC			

### Real-time PCR and expression of functional genes

Extracted total RNA was treated with DNase I (Fermentas, Life Sciences, Hannover, USA) as recommended by the manufacturer, followed by reverse transcription using the SuperScript™ III First Strand Synthesis System (Invitrogen, Carlsbad, USA), with random hexamer primers in accordance to the manufacturer's guidance. Real-time PCR was performed on *amoA*, *nxrA/B*, *narG*, *nirS*, and *nosZ* genes. Primers for these genes were designed in this study (Table 2), except for the *nirS* gene, for which the primers nirS1F and nirS3R (Braker et al., 1998) were used. Real-time PCR reactions were performed using the Maxima™ SYBR Green qPCR Master Mix System (Fermentas, Life Science Inc., Hanover, USA) in a total volume of 25 μL containing 12.5 μL Maxima™ SYBR Green qPCR Master Mix, 0.5 μL of each primer, 1–3 μL of cDNA (depending on the target gene) and dH_2_O up to a final volume of 25 μL. Amplification of the real-time PCR products and melting curve analysis was carried out with a Corbett Rotor-Gene 6000 thermocycler, combined with the Rotor-Gene 6000 Series software 1.7.34. Conditions were as follows: initial denaturation at 95°C for 10 min, denaturation at 95°C for 30 s, annealing at 57°C (for primer pair narG-2F-RT/narG-2R-RT), at 58°C (for primers: Cren-amoA-TS-1F/Cren-amoA-TS-1R; nirS1F/nirS3R) and 60°C (for primers nxrA-RT-F/nxrA-RT-R, nxrB-LRT-F/nxrB-LRT-R, nosZ-1F-RT/nosZ-2R-RT), elongation at 72°C for 30 s, number of cycles: 40.

**Table 2:** **Primer designed for real-time PCR on functional genes**.

**Primer**	**Sequence 5′ to 3′**	**Target gene fragment**	**Fragment, length [bp]**	**References**
Cren-amoA-TS-1F	GAT CTC ACA GTC TAC GAT	Archaeal *amoA*	203	This study
Cren-amoA-TS-1R	ACG AGA GGT CCA GCA GCA			This study
nxrA-RT-F	GTG GTC ATG CGC GTT GAG CA	*nxrA Nitrospira*	197	This study
nxrA-RT-R	TCG GGA GCG CCA TCA TCC AT			This study
nxrB-LRT-F	GGC AAT TGG GAC GGA AGA T	*nxrB Nitrospira*	184	This study
nxrB-LRT-R	TAG GGC TTG GTC TCC ACG T			This study
narG-2F-RT	ACG TCG AGA CCA ATC CGC TG	*narG Burkholderiales*	151	This study
narG-2R-RT	AAC AGG TTG CGC GGA AAG TT			This study
nosZ-1F-RT	GCT GAC CAA CGA GTC GA	*nosZ*	196	This study
nosZ-2R-RT	GAC GAT CTT GTC GCA CTC			This study

Real-time PCR products were checked on agarose gels for the correct size, excised and purified as described above. Sequencing of real-time PCR products was performed by the labs of Eurofins mwg/operon (Ebersberg, Germany), using the forward and reverse primers listed in Table 2, according to the guidance of the company. Similarity searches were carried out with the FASTA program and in addition with the BLAST® program on the web interface of the National Center for Biotechnology Information (NCBI).

### Phylogenetic analysis

Phylogenetic analyses were performed on the archaeal *amoA* as well as on bacterial *nxrA/B*, *narG*, *nirS*, *cnorb/qnorB*, and *nosZ* genes using the program MEGA5 (Tamura et al., 2011). Calculations for the archaeal *amoA* gene were based on up to 640 corresponding positions (including shorter sequences of 256 bp). Calculations on bacterial genes were based on 226 deduced amino acid positions for the *nxrA* and on 413 amino acid positions for the *nxrB* gene (including shorter sequences of 163 amino acids). The calculations of DNA sequences of N-metabolizing genes were based on the following homologous nucleotide positions: 650 for the *narG*, 881 for the *nirS*, 450 for the *cnorB* and 260 for the *qnorB* gene, and 1152 positions for the *nosZ* gene.

For each gene, or deduced amino acid sequence, the calculations were performed using the methods of neighbor-joining (NJ), maximum-parsimony (MP), and maximum-likelihood (ML), each with 100 replicates, while gaps were excluded from the analysis. Tree topology for all genes/deduced amino acids was based on the NJ method using the Jukes-Cantor distances, while bootstrap values were displayed as indicated in the figures.

### Nucleotide sequence accession numbers

Functional gene sequences recovered in this study were deposited at the European Nucleotide Archive (ENA) database with the following accession numbers: HF936730–HF936751 (for standard PCR sequences), HF936752–HF936762 (for real-time PCR sequences).

## Results

### Sampling site and properties of the thermal mineral spring

Pictures from the subsurface water discharges of the FJQ, where the samples were obtained, were shown in Weidler et al. (2007). Additional photos of the site are available in Gerbl (2013). The spring water had a temperature of 43.6°C at a pH of about 8. *In situ* measurements of the O_2_ content revealed up to 1.9 mg L^−1^ oxygen dissolved in the thermal water of the FJQ. The chemical composition of the spring water of the FJQ was similar to that described seven years ago by Weidler et al. (2007) and consisted of cations (in brackets: mg L^−1^ of thermal water): Na^+^ (76.0), Ca^2+^ (19.6), NH^+^_4_ (up to 0.02), Mn^2+^ (traces); anions: SO^2−^_4_ (130.0), HCO^3−^ (60.8), Cl^−^ (26.0), NO^3−^ (up to 0.5), NO^2−^ (up to 0.003), F^−^ (traces).

### Chemolithotrophic ammonia-oxidation and ^15^N-compounds

**Figure 1:**
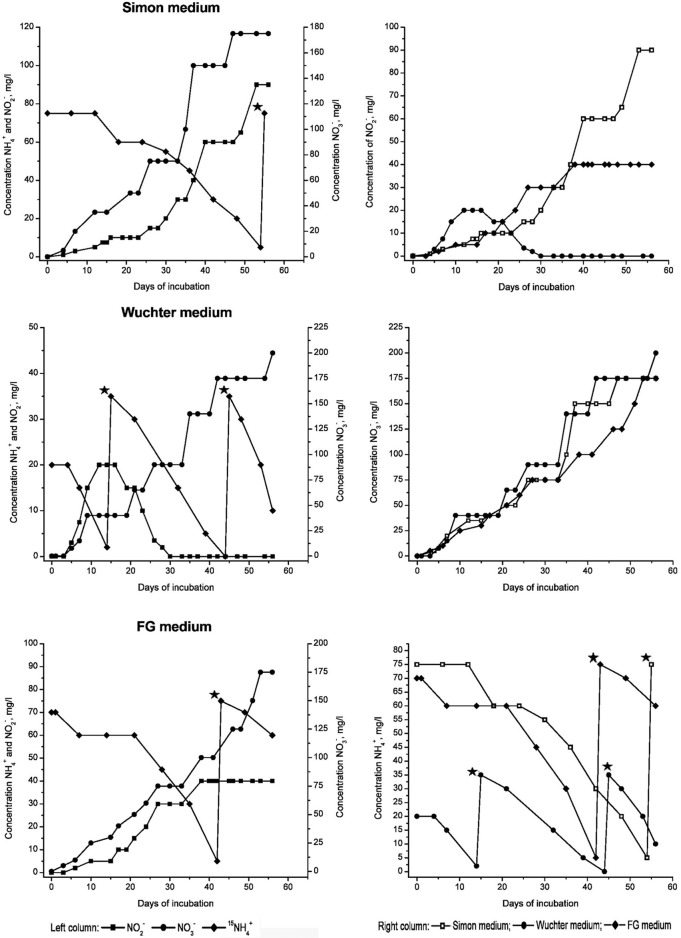
**Oxidation of ammonium and formation of nitrite and nitrate in microcosms, which were prepared with Simon, Wuchter, or FG medium, respectively, and labeled nitrogen compounds (left panels)**. The inoculum consisted of filtered (planktonic) microbes from the FJQ spring. Incubation temperature was 40°C. Substrates (^15^NH_4_Cl or [^15^NH_4_]_2_SO_4_, depending on the medium) were added if less then 10 mg/L NH^+^_4_ was measured. Time of addition is indicated by asterisks (✭). Panels on the right show summaries of the formation of nitrite, nitrate, and the decrease of ammonium, respectively, (from top to bottom).

Three different media—Wuchter, Simon, and FG medium—were used to find the optimal composition of nutrient supplements supporting growth of the microbial community inhabiting the FJQ in microcosms. All media were based on the thermal mineral spring water which was filter-sterilized and subsequently supplements were added (see Materials and Methods). Microcosm experiments of several weeks in duration showed a decline of ammonium that correlated with an increase of nitrite and nitrate (Figure 1). Control experiments without inocula were performed to ensure that ammonium oxidation as well as nitrite oxidation and the formation of nitrate were caused only by microbial metabolic activity. No alterations of the initial amounts of ammonium were observed during the incubation for at least 315 days and no formation of nitrite or nitrate, respectively, was detected in the control experiments (data not shown). The anaerobic (oxygen-limited) microcosms showed formation of large gas bubbles starting after 8–9 days of incubation (Gerbl, 2013). All three media apparently fulfilled the requirements of enriching the microbial community of the FJQ, although possibly different parts of it. Slight differences were observed between the three media, the incubation temperature (30 or 40°C, respectively) as well as the inocula used, which were either concentrated planktonic microorganisms or biofilms from the rocks surrounding the water discharges. Figure 1 shows one example of the microcosm experiments, which contained FG medium, an inoculum of planktonic microorganisms and was incubated at 40°C.

**Figure 2:**
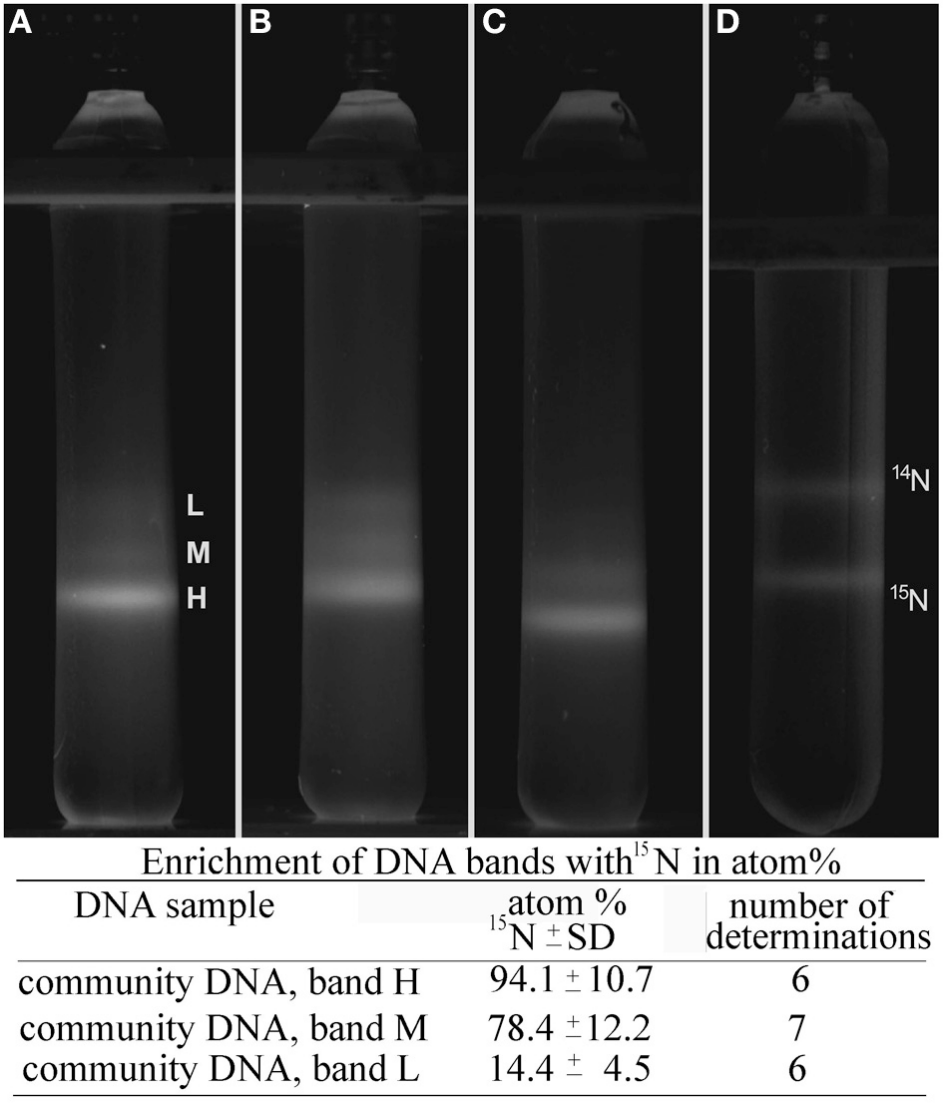
**Isopycnic centrifugation of ^15^N labeled community DNA in CsCl/ethidium bromide density gradients**. Centrifuge tubes were exposed to a UV transilluminator and photographed. **(A–C)** DNA extracted from microcosm made with FG medium **(A)**, Wuchter medium **(B)**, or Simon medium **(C)**, respectively, inoculated with planctonic microorganisms **(A,C)** or biofilm material **(B)**. Incubation temperature was 40°C for all microcosms. **(D)** A mixture of labeled (^15^N) and unlabeled (^14^N) DNA, extracted from *Nitrosomonas europaea*. L, M, H in **(A)** indicate the positions of light, medium, and heavy DNA bands, respectively. The enrichment of DNA bands with ^15^N in atom %, as determined by isotope ratio mass spectrometry (IRMS), is indicated.

For SIP substrates labeled with ^15^N (either [^15^NH_4_]_2_SO_4_ or ^15^NH_4_Cl) were added to microcosms depending on the medium used. During metabolic activity the label is being incorporated into the biomass (DNA) of the microbes metabolizing ammonium, nitrite or nitrate, increasing the density of DNA, which can then be separated from unlabeled DNA (Buckley et al., 2007; Cupples et al., 2007). For the current study ^15^N-labeled nitrogen compounds were used as sole energy sources. Successful incorporation of the heavy nitrogen isotope into the DNA of members of the microbial community was confirmed by comparing the positions of bands of DNA extracted from microcosms after isopycnic centrifugation (Figure 2). The density of the band of DNA extracted from microcosms supplemented with unlabeled substrates was determined from its position in the gradient as 1.60–1.61 g mL^−1^ (Figure 2, band L), whereas ^15^N-labeled DNA was located at densities of 1.62–1.63 g mL^−1^ (Figure 2, bands M and H). These shifts in buoyant density corresponded well to results published earlier for DNA-SIP, using labeled nitrogen compounds (Cadisch et al., 2005; Buckley et al., 2007; Cupples et al., 2007). Control experiments with DNA extracted from pure cultures of *Nitrosomonas europaea*, an ammonia-oxidizing chemolithoautotrophic bacterium grown in mineral media supplemented either with ^14^NH_4_Cl or ^15^NH_4_Cl, showed the incorporation of the label into its DNA (Figure 2D). The incorporation of labeled nitrogen molecules into biomass (DNA) of members of the thermal mineral spring FJQ was confirmed by IRMS (Figure 2), which revealed ^15^N enrichments of the heavy DNA band of 94.1 at%. This finding agreed with results published by Cadisch et al. (2005), which stated a ^15^N enrichment up to 90 at% for DNA of an environmental microbial community. Some contamination during recovery of bands likely took place, since the at% of band L is somewhat higher than expected.

The ^15^N-labeled DNA from microcosms was also used for the phylogenetic identification of microbial community members in the FJQ, which can utilize nitrogen compounds, and thus turn the wheel of a putative nitrogen cycle (data not shown; manuscript in preparation).

### Probing for functional genes involved in nitrogen transformations

Two approaches were used: genes coding for proteins involved in nitrogen cycling were searched for with crude extracts of genomic DNA from the microcosms. Genes *amoA*, *nxrA*, *nxrB* were amplified from DNA of aerobic microcosms, genes *nirK*, *nirS*, *qnorB*, *cnorB*, *napA*, *narG*, and *nosZ* from DNA of oxygen-limited microcosms. In addition, real-time PCR experiments with cDNA obtained from reverse transcriptase reactions were carried out with total RNA extracts from the microcosms to prove that these genes were actually transcribed in the microcosm experiments. Primer pairs from various environmental studies (see Table 1) were tested for the amplification of genes coding for functional key enzymes. Primer pairs for the genes *nifH/D* were selected from references Zani et al. (2000); Minerdi et al. (2001); Poly et al. (2001), and Zehr et al. (2003) and used under the conditions described by these authors. PCR-products of the expected size were cloned as fragments of the genes *amoA*, *nxrA*, *nxrB*, *nirK*, *nirS*, *qnorB*, *cnorB*, *napA*, *narG*, *nosZ* as well as for the gene *nifH*. Nucleic acid sequences of the investigated genes were compared to entries in both GenBank and EMBL-EBI databases, using blastn and FASTA search tools, respectively. Nucleotide sequences were translated into protein sequences using the Translate tool on the ExPASy (Expert Protein Analysis System) proteomics server of the Swiss Institute of Bioinformatics (http://us.expasy.org/tools/dna.html). Deduced amino acid sequences were also subjected to database searches for related sequences at GenBank (blastp) and EMBL-EBI. Table S1 (Supplementary Material) lists the closest relatives obtained from the database (EMBL-EBI).

#### AmoA genes (ammonium oxidase, subunit A)

**Figure 3:**
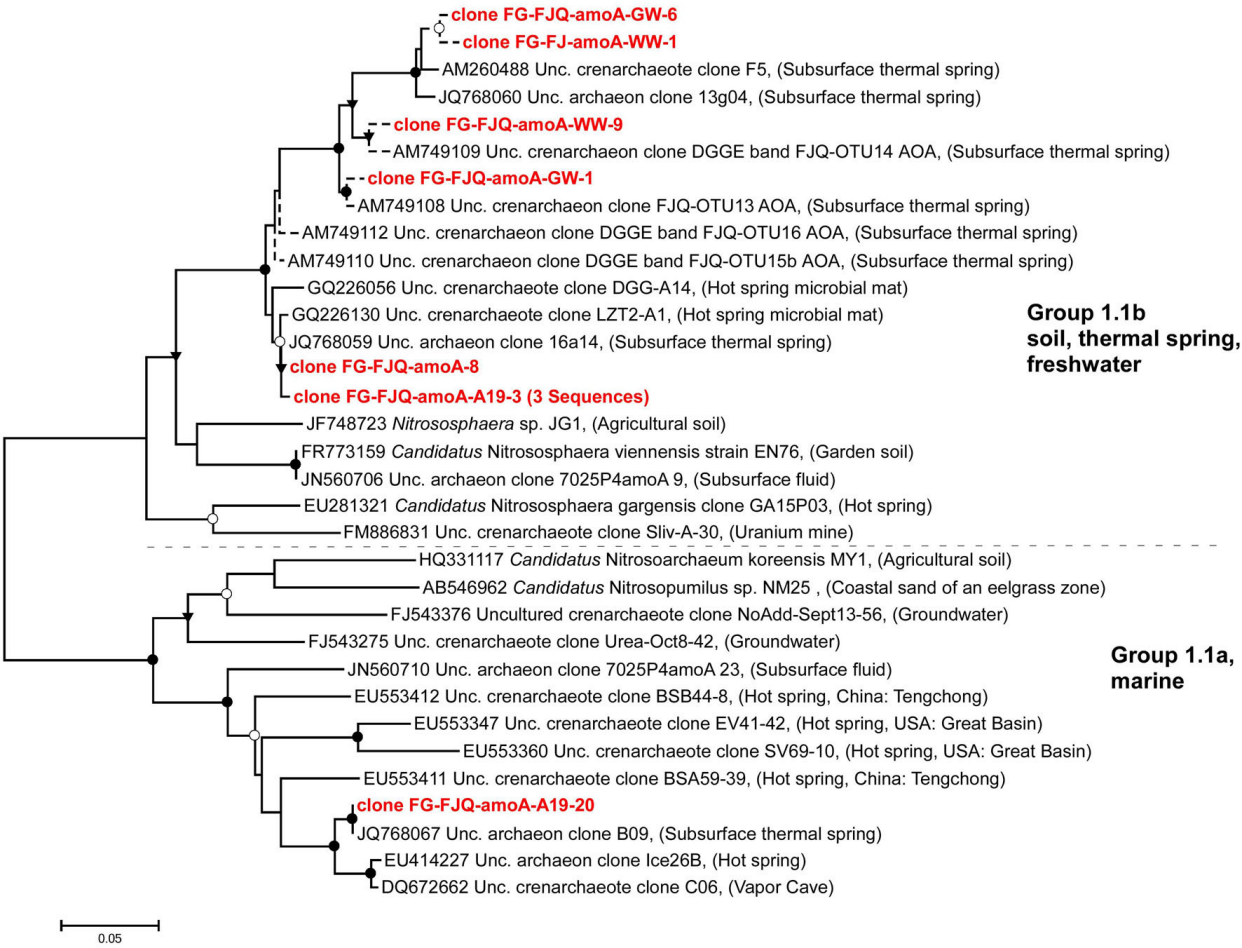
**Phylogenetic relationships among *amoA* sequences from FJQ microcosms and closest database sequences**. Short sequences (256 bp) are represented by dotted lines and did not change the overall topology of the tree. The tree was generated using the neighbor-joining method (NJ) and topology was supported by maximum-likelihood as well as maximum-parsimony methods. Bootstrap values (NJ) from 100 replicates were indicated as follows: ●, >90%; ○, 70–89%; ▾, 50–69%. Scale bar indicates substitutions per site.

The phylogenetic analysis of the *amoA* sequences obtained in this study is shown in Figure 3. Two main clusters, which had been previously identified, were recognizable, group 1.1a (marine/sediment/freshwater) and group 1.1b (soil/freshwater/thermal spring). Recently up to 5 clusters were suggested by Pester et al. (2012), together with a new nomenclature. Clone FG-FJQ-amoA-8 stemmed from microcosm set up with Wuchter medium, clones FG-FJQ-amoA-A19-3 and FG-FJQ-amoA-A19-20 from microcosms with Simon medium. These sequences were amplified with the primer pair CrenAmo1F and CrenAmo1R (Könneke et al., 2005) producing an amplicon of 640 bp. The clones FG-FJQ-amoA-8 and FG-FJQ-amoA-A19-3 clustered within group 1.1b and showed up to 100% sequence identity to an uncultured archaeon clone 16a14 (JQ768059) generated during a metagenomic study on the spring environment of the FJQ (Bartossek et al., 2012). They were about 99% similar to clones GQ226056 and GQ 226130 from hot springs in China (Jiang et al., 2010). They exhibited 90% identity to the *amoA* gene of the closest related cultured archaeon, *Candidatus* Nitrososphaera sp. strain EN76 (FR773159) and to a clone (JN560706) from deep borehole fluids of a temperature of 40°C (Swanner and Templeton, 2011). A relationship of 87 and 83% similarity to *Candidatus* Nitrososphaera gargensis (EU281321) and a partial *amoA* sequence, respectively, from an uranium mine (FM886831; Radeva et al., 2014) existed to clones FG-FJQ-amoA-8 and FG-FJQ-amoA-A19-3. Five clones with a sequence length of 256 bp (including 2 identical sequences), obtained with primer pair Arch-amoA-F/Arch-amoA-R, fell into group 1.1b, and all nearest *amoA* database sequences represented uncultured thaumarchaeal/archaeal clones from the spring FJQ (sequence identities between 97.7 and 98.8%).

Clone FG-FJQ-amoA-A19-20 clustered among group 1.1a and had 100% sequence identity to an uncultured archaeal clone B09 (JQ768067) from the metagenomic study mentioned above (Bartossek et al., 2012). The *amoA* gene fragments of the next cultured relatives *Nitrosopumilus maritimus* SCM1 (EU239959; Reigstad et al., 2008; data not shown) and *Candidatus* Nitrosoarchaeum koreeinsis MY1 (HQ331117) both showed 82.7% sequence identity to clone FG-FJQ-amoA-19A-20. Another clone (JN560710) from the borehole fluid (Swanner and Templeton, 2011) was 89% similar. Clones EU553347, EU553360, and EU553411 from terrestrial hot springs in China, Russia and USA (Zhang et al., 2008) were 90, 91, and 85% similar. Two clones (FJ543275, FJ543376) from a low nutrient aquifer in Idaho, USA (Reed et al., 2010) were 86 and 80% similar. The phylogenetic relationship to the closest cultured relatives, *Candidatus* Nitrososphaera sp. strain EN76 and *Nitrososphaera* sp. strain JG1 (JF478723) was of 81.6–85.5% sequence identity.

#### NxrA and NxrB genes (nitrite oxidoreductase genes, subunit A and subunit B)

**Figure 4:**
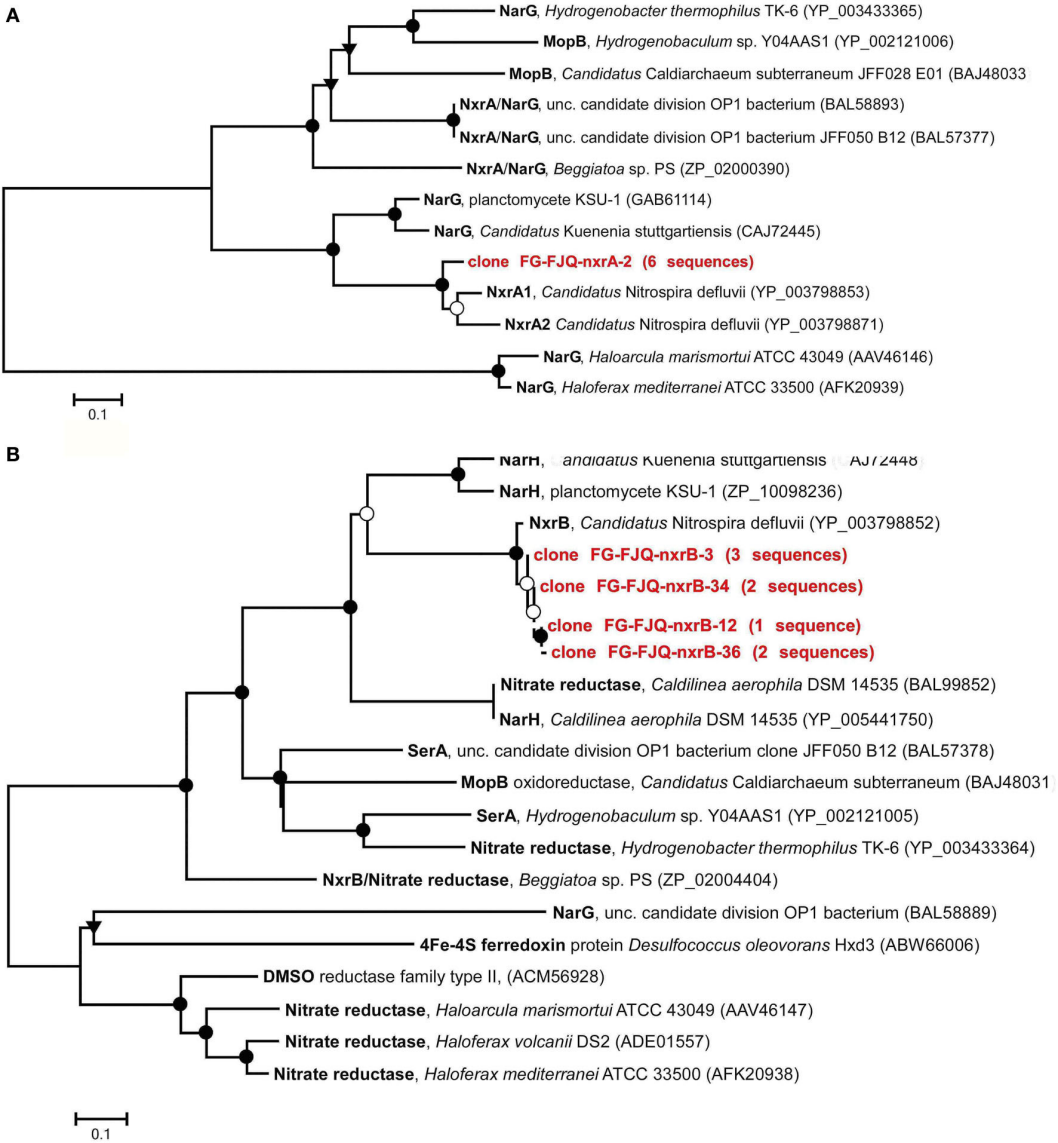
**Phylogenetic relationships within the molybdopterin-binding protein superfamily. (A)** Dendrogram derived from the deduced protein sequence (226 amino acids) encoded by the *nxrA* gene and closest related sequences for alpha subunits of the subfamily of enzymes within the MopB protein superfamily. Abbreviations: NxrA, nitrite oxidoreductase alpha subunit; NarG, membrane bound respiratory nitrite reductase; MopB, molybdopterin oxidoreductase. **(B)** Dendrogram derived from the deduced protein sequences (413 amino acids and 163 amino acids, respectively; the latter are indicated by dotted lines) encoded by *nxrB* gene. Sequences of the molybdopterin-binding proteins (MopB) reflecting the closest relatives obtained from databases are shown. Abbreviations: NxrB, nitrite oxidoreductase beta subunit; NarH, membrane bound nitrate reductase beta subunit; SerA, selenate reductase; DMSO, dimethyl-sulfoxide. Further details are as described in the legend to Figure 3. Scale bars indicate substitutions per site.

The continuous increase of the concentration of nitrate (Figure 1) during incubation of microcosms suggested the presence of nitrite-oxidizing *Bacteria* (NOB). Phylogenetic analysis of the 16S rRNA gene fragments was in agreement with this notion, since clones belonging to the *Nitrospira* class were found (Weidler et al., 2007; Gerbl, unpublished data). Primers targeting the genes encoding for the nitrite-oxidoreductase (*nxrA* and *nxrB*), an enzyme which carries out the process of NO2^−^ oxidation to NO3^−^, were described by Maixner (2009) and Lücker et al. (2010) and used for amplification with PCR. The products of both *nxrA* as well as *nxrB* genes were cloned and sequenced. However, phylogenetic reconstructions using these nucleotide sequences resulted in dendrograms which were difficult to interpret. Therefore, the nucleotide sequences were translated into amino acid sequences. Deduced amino acid sequences were then subjected again to database searches for related protein sequences at GenBank (blastp) and EMBL-EBI. Six randomly chosen clones were obtained which had all the same nucleotide sequence and therefore clone FG-FJQ-nxrA-2 was selected as representative of the *nxrA* gene sequences. Figure 4A shows that the NxrA1 and NxrA2 sequences of *Candidatus* Nitrospira defluvii (YP003798853 and YP003798871) were the closest relatives (88.1 and 88.5% sequence identity) of clone FG-FJQ-nxrA-2. Protein sequences of the membrane bound respiratory nitrate reductase alpha subunit (NarG), which belongs to the molybdopterin oxidoreductase superfamily, derived from *Candidatus* Kuenenia stuttgartiensis and the planctomycete strain KSU-1 then followed with 66.8 and 68.5% sequence identity.

Clones created for subunit B of the nitrite oxidoreductase showed slight differences of about 1% in nucleotide sequences. The phylogenetic relationships of the deduced protein sequences of the clones FG-FJQ-nxrB-3, FGFJQ-nxrB-34, FG-FJQ-nxrB-12 and FG-FJQ-nxrB-36 are shown in Figure 4B and in Table S1. The protein sequence for the NxrB protein of *Candidatus* Nitrospira defluvii (YP003798852) was the closest related neighbor with 96.0–96.4% sequence identity for the clones with 413 amino acid positions (clones FG-FJQ-nxrB-3 and FG-FJQ-nxrB-34) and 94.0–95% for the protein sequences with 163 amino acid positions (clone FG-FJQ-nxrB-12 and clone FG-FJQ-nxrB-36). All of the database relatives belonged to the DMSO reductase type II family.

#### NarG genes (membrane bound respiratory nitrate reductase, alpha subunit)

The membrane bound nitrate reductase (Nar) carries out the first step in respiratory denitrification, the reduction of nitrate (NO^−^_3_) to nitrite (NO^−^_2_) and is encoded by the genes of the *narGHJI* operon. The *narG* gene encodes the subunit A of this enzyme and has been widely used as a marker gene for environmental studies (Chèneby et al., 2003; Gregory et al., 2003; Smith et al., 2007).

**Figure 5:**
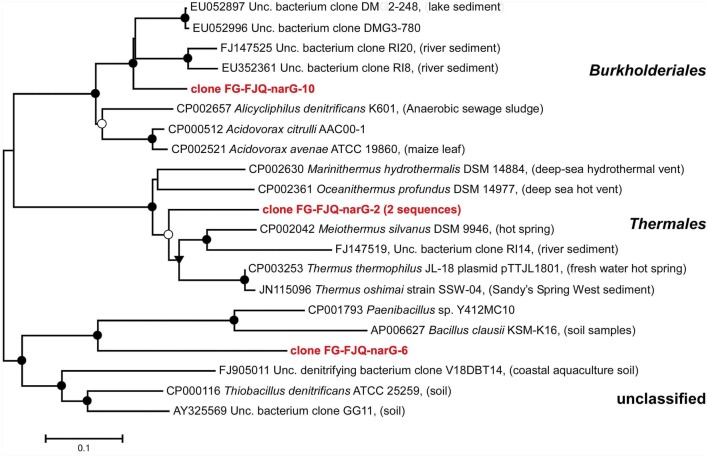
**Phylogenetic relationships of the *narG* sequences obtained from microcosms of the spring FJQ to the closest related *narG* gene sequences from databases**. The tree was inferred by a neighbor-joining analysis of 650 nucleotide positions. Further details are as described in the legend to Figure 3.

Using primer pair narG1960F/narG2650R led to five randomly selected clones which comprised three different nucleotide sequences. The phylogenetic affiliation of the sequences for the clones FG-FJQ-narG-2, FG-FJQ-narG-6 and FG-FJQ-narG-10 is shown in Figure 5. Clone FG-FJQ-narG-2 belongs to the *nar-genes* of the order of the *Thermales* and showed sequence identities of 80.3% to the closest related neighbor in the database, nitrate reductase alpha subunit from *Meiothermus silvanus* DSM 9946 (CP002042), which was isolated from a hot spring. Database search for clone FG-FJQ-narG-10 revealed affiliation of this phylotype to the *Burkholderiales*, with the *narG* gene of the uncultured bacterium clone DMG2-248 (EU052897) as closest relative sequence with a sequence identity of 87.4%. One of the closest neighbor sequence obtained from a cultured organism was the sequence for a nitrate reductase alpha subunit of *Alicycliphilus denitrificans* K601 (CP002657), which showed 81.2% sequence similarity. Clone FG-FJQ-narG-6 was distantly related to the next database neighbor *Paenibacillus* sp. Y412MC10 (CP001793) with only 62.5% sequence identity (see Table S1), followed by the nitrate reductase alpha subunit of *Thiobacillus denitrificans* ATCC 25259 with 61.2% sequence identity. Since the *Paenibacillus* sp. Y412MC10 belongs to the phylum *Firmicutes* and *Thiobacillus denitrificans* ATCC 25259 represents the phylum *Betaproteobacteria*, the sequence of clone FG-FJQ-narG6 remained unclassified.

A second type of nitrate reductase is the periplasmatic enzyme Nap. No PCR-products of the expected size could be amplified with the primers used by Henry et al. (2008).

#### NirS gene (cd1-type nitrite reductase gene)

Nitrite reductases are key enzymes in the dissimilatory denitrification process, driving the catalysis of nitrite reduction to nitric oxide (NO^−^_2_ to NO). Two classes of nitrite reductase enzymes exist, which are distinguished by their cofactors cytochrome (cd1Nir) and copper (CuNir). Only one type of nitrite reductase gene could be recovered successfully via PCR-amplification. Two primer sets developed by Braker et al. (1998), spanning different length of the *nirS* gene (encoding for cd1Nir), were used. After successful amplification and cloning, five clones from the clone library created with the PCR-products of about 890 bp in length and two clones from the clone library with the shorter PCR-products (257 bp) were randomly selected and sequenced.

**Figure 6:**
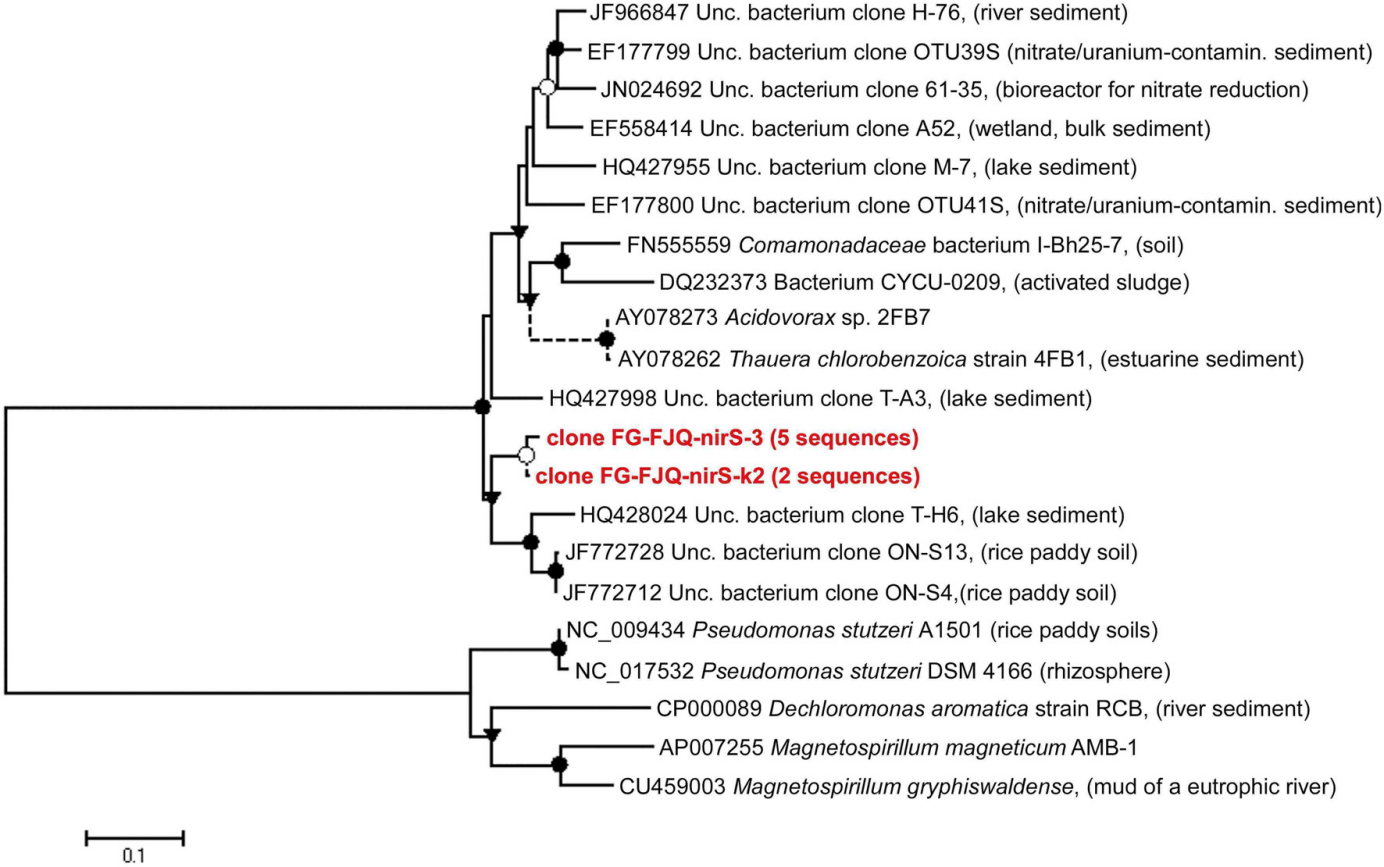
**Phylogenetic relationships of *nirS* gene sequences obtained from microcosms of the spring FJQ**. The tree was generated by neighbor-joining analysis of 881 nucleotide positions. Short sequences (257 bp) are indicated by dotted lines and did not change the topology of the tree. Further details are as described in the legend to Figure 3.

The phylogenetic relationship of the clones to related sequences for *nirS* genes is displayed in Figure 6 and Table S1. Sequence alignments showed that the closest related database *nirS* gene sequence for clone FG-FJQ-nirS-3 was that from an uncultured bacterium, clone T-H6 (HQ428024), which was recovered from lake sediment. For clone FG-FJQ-nirS-k2, representing the short *nirS* gene sequence fragments (257 bp), the uncultured bacterium clone ON-S4 *nirS* gene (JF772712) isolated from rice paddy soil was the next similar sequence recovered from the database.

#### CnorB and qnorB (nitric oxide reductases genes, large subunit)

Under oxygen-limited conditions when oxidized nitrogen compounds are used as alternative electron acceptors for microbial respiratory denitrification, NO is produced as an intermediate (Carr et al., 1989; Zumft, 1997). NO in excess is toxic and NO reductases have either a detoxifying role (de Vries et al., 2007), or are part of the denitrification chain for energy-conserving for growth (Zumft, 1997). There are different types of NO reductases in microorganisms. Primer sets for a gene fragment encoding the cytochrome *c*-containing cNorB, or for a gene fragment encoding the quinol dependent qNorB protein were used (see Table 1). PCR-products of the expected sizes (260 bp for qnorB and 450 bp for cnorB) were obtained, however, sequences of most clones were not of the right size and yielded meaningless database records.

**Figure 7:**
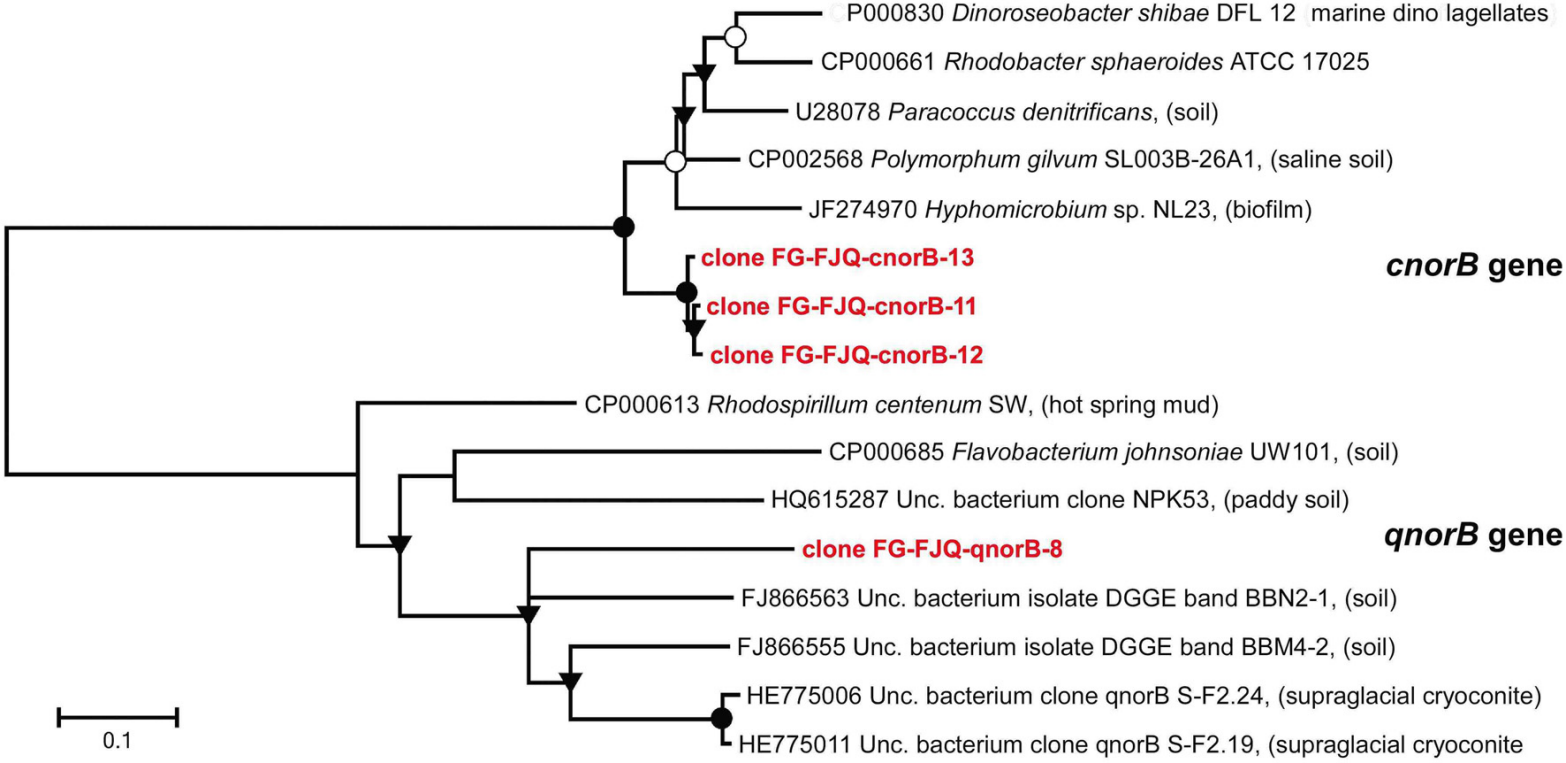
**Phylogenetic relationships of *cnorB* and *qnorB* gene fragments obtained from microcosms of the spring FJQ**. The tree was generated by neighbor-joining analysis of 450 nucleotide positions of the *cnorB* gene and of 260 nucleotide positions of the *qnorB* gene. Further details are as described in the legend to Figure 3.

Three clones for *cnorB* gene fragments and only one clone for *cnorB* gene fragments were successfully sequenced (Figure 7). All three clones for the fragment of the *cnorB* gene were only slightly different in nucleotide sequence (98.7–99% sequence similarity). However, the closest neighbors from database searches were the gene from *Polymorphum gilvum* SL003B-26A1 (CP002568) (83–84% sequence identity) and the *cnorB* gene of an uncultured bacterium clone norB9 (JN559463) (83–84% similarity), isolated from a biofilm in a denitrification system; those are listed in Table S1.

The relatedness of the sequence of the one clone for the *qnorB* gene to its next neighbors was considerably lower. Clone FG-FJQ-qnorB-8 showed sequence similarities of only 71% to the closest related sequence from environmental databases, a *qnorB* sequence from an uncultured bacterium (FJ866555). The next neighbors from cultured databases were *qnorB* gene sequences from *Rhodospirillum centenum* (CP000613) and *Flavobacterium johnsoniae* (CO000685), which were both of about 63% sequence similarity.

#### NosZ-genes (nitrous oxide reductase genes)

Nitrous oxide is not only produced as an intermediate of denitrifying prokaryotes, it is also generated by metabolic activity of nitrifiers, methanotrophic bacteria and fungi. But only the denitrifying microorganisms possess the genetic inventory to convert N_2_O into N_2_ and use this for energy-conservation (Zumft, 1997; Zumft and Körner, 2007). The *nosZ*-gene encodes the catalytic subunit of the nitrous oxide reductase and is therefore suitable as a functional marker gene for environmental studies (Henry et al., 2006).

**Figure 8:**
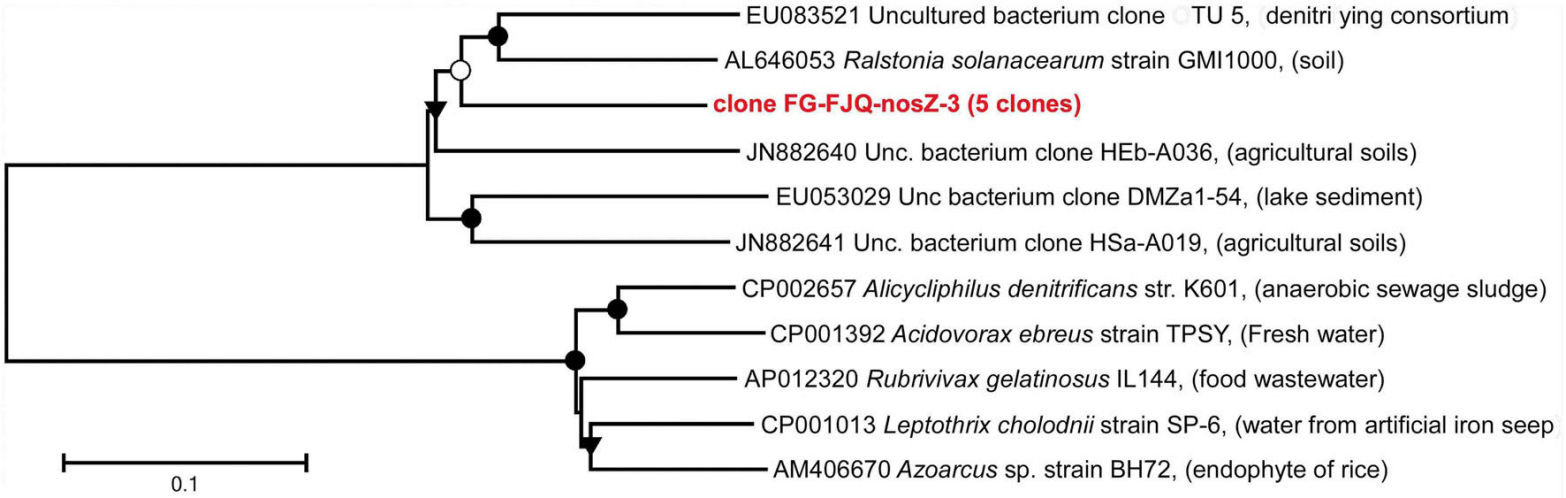
**Phylogenetic relationships of clones for *nosZ* gene gene fragments**. The tree was generated by neighbor-joining analysis of 1152 nucleotide positions. Further details are as described in the legend to Figure 3.

Five clones were obtained using primers for the *nosZ* gene, which were of the same nucleotide sequence. The phylogenetic affiliation of these five clones is such that the gene for nitrous oxide reductase from *Alicycliphilus denitrificans* strain K601 (CP002657), isolated from anaerobic sewage sludge was the closest relative form database search, showing 82.0% sequence identity. The most closely related sequence from environmental databases was the *nosZ* gene from the uncultured bacterium clone OTU 5 (EU083521), discovered from a denitrifying consortium (Figure 8; Table S1).

#### Nif genes (dinitrogenase genes)

Although several primer pairs (Zani et al., 2000; Minerdi et al., 2001; Poly et al., 2001; Zehr et al., 2003) were used for amplification of one of the key genes in the nitrogen cycle, the *nif* gene, especially the fragments *nifH* or *nifD*, no PCR product of the right size could be generated either for *nifH* or for *nifD*.

### Transcriptional analysis of genes involved in N-cycling

To prove that the genes involved in the nitrogen cycle are really expressed in microcosm systems under suitable conditions and hence responsible for the oxidation or reduction of the respective N-compounds, aerobic, and anaerobic (oxygen-limited) microcosms were prepared (see Materials and Methods, and Table 3). Reverse transcription of extracted total RNA was carried out for the generation of cDNA, which was subsequently used as template for real-time PCR experiments. Six selected gene fragments from nitrogen metabolizing enzymes were successfully amplified by real-time PCR (Table 3).

**Table 3:** **Nearest database sequences of N-metabolizing gene sequences obtained by real-time PCR**.

**Microcosm type and real-time PCR sequence**	**Target gene fragment**	**Fragment length [bp]**	**Closest database sequence**	**Accession number**	**Origin**
FG60_Cren-amoA	crenarchaeal *amoA*	203	Uncultured crenarchaeote clone F5	AM260488	Thermal spring
FG61_nxrA	*nxrA*	197	*Candidatus* Nitrospira defluvii, nxrA1	FP929003; Location: 3191005–3191680	Activated sludge
FG61_nxrB	*nxrB Nitrospira*	184	*Candidatus* Nitrospira defluvii, *nxrB1*	FP929003; Location: 3187008–3188297	Activated sludge
FG63-2_narG	*narG Burkholderiales*	151	*Comamonas nitrativorans* DSM 13191T, *narG* gene	AM419044	Denitrifying reactor
FG62-2_nirS	*nirS*	257	*Magnetospirillim magneticum* AMB-1, nitrite reductase	AP007255; Location: 1520665–1520921	Fresh water
FG62-2_nosZ	*nosZ*	196	*Rubrivivax gelatinosus* IL144, *nosZ* gene	AP012320; Location: 2007768–2007958	Food wastewater

*Microcosms types were: FG60, aerobic, FG medium, inoculum biofilm; FG61, Simon medium, inoculum planktonic microorganisms; FG63-2, anaerobic, FG medium, inoculum planktonic microorganisms; FG62-2, anaerobic, Wuchter medium, inoculum planktonic microorganisms. Incubation temperature was 40°C for all microcosms*.

Real-time PCR yielded a distinct PCR-product of the expected size (203 bp) of the crenarchaeal *amoA* gene fragment with cDNA generated from total RNA extracted from microcosms supplemented with FG medium. However, for the cDNA obtained from microcosms with Wuchter medium no amplicon could be generated with the primer pair used here. The sequence of the cDNA dependent crenarchaeal *amoA* PCR-product matched the sequences of the uncultured archaeal clone 17g13 (JQ768061) with 99% (FG 60) and 100% (FG 63). Clone 17g13 was recovered during a metagenomic study performed on the habitat of the spring FJQ (Bartossek et al., 2012). The closest database neighbors are listed in Table 3.

For both the *nxrA* and *nxrB* genes the targeted fragments were successfully amplified. Database searches with nucleotide sequences obtained from PCR-products revealed the putative oxidoreductase, alpha subunit (*nxrA*1) from *Candidatus* Nitrospira defluvii (FP929003) as closest relative (84% identities) for the *nxrA* gene sequence from microcosm FG 61 (Table 3). The sequence for the beta subunit (*nxrB*) of *Candidatus* Nitrospira defluvii (FP929003) turned out to be the nearest neighbor (91% identities) to the nucleotide sequence from the real-time PCR approach on the *nxrB* genes, also from microcosm FG 61.

Distinct PCR-products were obtained for the partial *narG* gene sequence. *Comamonas nitrativorans* (AM419044) was the nearest neighbor (73.3% identity) obtained from database search for cultured prokaryotes, and the partial *narG* sequence from clone C11_42 (FN430451) from environmental samples showed 80.0% identities. These results were the same for both Wuchter medium and FG medium microcosms.

Comparisons of the sequence against databases revealed a nitrite reductase precursor sequence from *Magnetospirillim magneticum* (AP007255) as closest relative from cultured microbes, showing 84.0% sequence identities (Table 3). From databases for environmental samples, the *nirS* gene sequence from clone OTU38S NirS (EF177798) was obtained as closest neighbor.

The closest relative of sequence FG62-2 nosZ was *Rubrivivax gelatinosus* (AP012320) with 84.3% sequence identities (see Table 3), while the sequence of FG63-2 nosZ showed only 81.7% identities. But both sequences met the *nosZ* gene sequence from *Acidovorax* sp. JS42 (CP000539) with the same percentage of 84.3%.

## Discussion

### Subsurface oxidation of ammonia and denitrification

Microbial communities of different environmental habitats are useful for understanding the cycling of the essential building blocks of life. The subsurface thermal mineral spring FJQ presents an easy to reach access to a subterranean environment, but without climatic or anthropogenic influence.

All three media used here for microcosms fulfilled the requirements of probably different parts of the microbial community of the FJQ. The values for NH^+^_4_ decreased in all microcosms while the values for NO^−^_2_ and NO^−^_3_ increased simultaneously. The consumed quantities of NH^+^_4_ did not differ significantly between the three media. All media lacked organic carbon sources, which could presumably be used for fermentation; thus energy conserving respiratory denitrification likely took place under anaerobic or oxygen limited conditions. In anaerobic microcosms the development of gases was noted, which likely consisted of NO, nitrous oxide and dinitrogen. This observation supported the presence of the respective enzymes (nitrite reductase, nitric oxide reductase, nitrous oxide reductase).

In this study ^15^N labeled nitrogen compounds were used to identify microbial communities, which utilize these compounds as sole energy source, and incorporate the isotopic label into their DNA (see below). The control experiments with microcosms performed with each of the three media, but supplemented with unlabeled (^14^N) nitrogen compounds, showed no significant differences of the measured quantities of consumed NH^+^_4_ and produced NO^−^_2_ or NO^−^_3_. Thus, the possibility of discriminating between ^15^N- and ^14^N- labeled nitrogen compounds by the microorganisms appeared unlikely.

### Stable isotope probing (SIP)

Successful incorporation of ^15^N into the DNA of members of the microbial community of the FJQ spring was confirmed (Figure 2). The distance between ^15^N-labeled DNA and unlabeled DNA was measured as 9–10 mm during all isopycnic centrifugation runs in this work. Cadisch et al. (2005) determined a distance of 5 mm between ^15^N labeled DNA and unlabeled DNA. The extended distance was likely due to the larger volume and length of the ultracentrifugation tubes. The influence of ethidium bromide on the buoyant density of the observed DNA was assumed to be negligible for the results of isopycnic centrifugation (Gallagher et al., 2010), since always the same amount of ethidium bromide was added to the samples.

### Thaumarchaeal *amoA* genes from other geothermal environments

It is now well established that ammonia oxidizing archaea occur over a wide range of temperatures from cold marine sediments to hot springs (Stahl and de la Torre, 2012). Several studies described thaumarchaeal *amoA*-like genes in moderately hot springs and other geothermal environments with temperatures of about 45–50°C (Spear et al., 2007; Reigstad et al., 2008; Zhang et al., 2008; Jiang et al., 2010; Swanner and Templeton, 2011; Zhao et al., 2011; Ragon et al., 2013). Sampling of springs often involves collection of mud, sediments and microbial mats (biofilms) at the outflows, in addition to concentration of spring water. The question of potential contamination of samples from the environmental surroundings has been considered by many authors. The *amoA* clones from the FJQ revealed remarkable similarity and sometimes identity of sequences to those from other samples of geothermal systems, whether liquid, solid, or semi-solid (Figure 3; Weidler et al., 2007; Jiang et al., 2010; Bartossek et al., 2012). This provided strong evidence for the notion that genuine geothermal microorganisms and not contaminants from the outside had been analyzed. Another interesting aspect is the finding of archaeal *amoA* genes with similarity to the FJQ clones in an uranium mine (Radeva et al., 2014), since the presence of radioactivity (radium, radon, and uranium) is characteristic of the thermal springs of Bad Gastein (Heinen and Lauwers, 1988).

### Genes involved in the N-cycle

**Figure 9:**
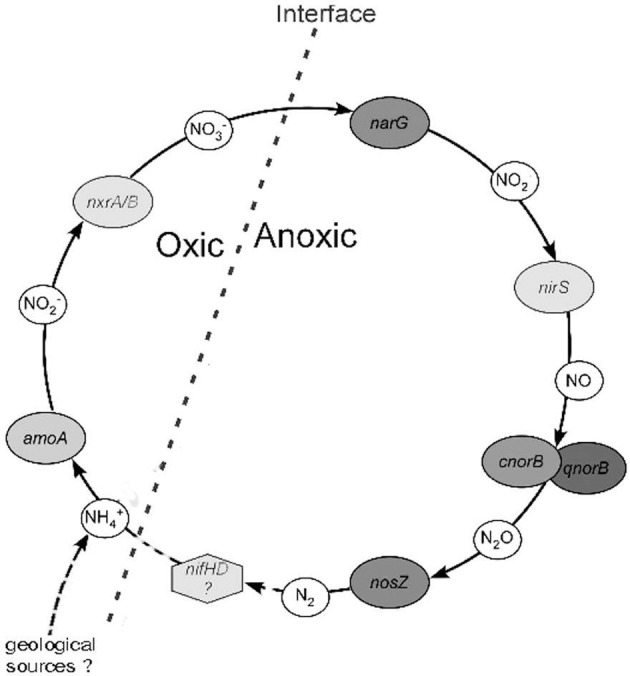
**Schematic representation of a proposed N-cycle in the FJQ spring**. The oxic-anoxic interface is indicated by a bold broken line. Genes or gene fragments identified in this work are shown in oval frames. NH^+^_4_ may derive from geological origins or via N_2_ fixation (*nif* genes), denoted by dashed lines.

Figure 9 shows a schematic representation of a possible N-cycle in the FJQ, based on the information obtained from genes or gene fragments. The first steps are carried out by the enzymes ammonium monooxygenase (Amo) and nitrite-oxide oxidoreductase (Nxr), which are encoded by the genes *amo* and *nxr*, respectively. The enzymes nitrate reductase (Nar), nitrite reductase (Nir), nitric oxide reductase (Nor) as well as the nitrous oxide reductase (Nos) are crucial for the reductive steps in nitrogen cycling. These proteins or their subunits are encoded by gene fragments of *narG*/*napA*, *nirS*/*nirK*, *cnorB*/*qnorB*, and *nosZ* (Braker et al., 2000; Philippot, 2002; Kandeler et al., 2006; Geets et al., 2007; Gruber and Galloway, 2008; Henry et al., 2008).

In this study genes or fragments of the genes *amoA*, *nxrA* and *nxrB*, *narG*, *nirS*, *cnorB* and *qnorB* as well as of the *nosZ* gene were successfully amplified, cloned and sequenced by standard PCR from DNA extracted from microbial community established in microcosms. No PCR-product for the bacterial encoded *amoA* gene could be amplified with the widely used primers for the *amoA* gene of the AOB of the *Betaproteobacteria*. This could perhaps be explained by the low oxygen concentration in the FJQ spring water, which might lead to outcompeting of AOB by AOA. The detection of *amoA* gene fragments of AOA and the apparent absence of bacterial *amoA* genes among the DNA from the microcosms suggested that the first step of nitrification in the spring environment is carried out by species belonging to the *Thaumarchaeota*, as was proposed previously (Weidler et al., 2008). The occurrence of NOB in the FJQ was already suggested by Weidler et al. (2007), since *Nitrospira* clones were found. Translated protein sequences of the nitrate reductase subunit A obtained here and subunit B of the nitrite oxidoreductase both were most similar to the respective sequences of *Candidatus* Nitrospira defluvii.

The presence of distinct functional genes does not necessarily imply functional activity of these genes within microbially driven ecosystems (Prosser and Nicol, 2008). Thus, reverse transcription of total RNA followed by real-time PCR experiments was used to show expression of the archaeal and bacterial nitrification and denitrification genes. Primers for real-time PCR were successfully designed in this work (Table 2) and gene fragments of *amoA*, *nxrA/B*, *narG*, *nirS* and *nosZ* were identified within microbial communities obtained from microcosm experiments. Thus, the standard PCR results obtained with DNA preparations from microcosms were corroborated with real-time PCR obtained from extracted RNA which was transcribed into cDNA and used as template.

Detailed phylogenetic studies of the microcosms revealed evidence for representatives of all orders and phyla implied in the FJQ communities—*Firmicutes, Thermales, Burkholderiales, Planctomyces, Rhodocyclales, Rhizobiales, Bacteriodetes*, and *Acidomicrobiales* (data not shown; manuscript in preparation).

### Nitrogen compounds from geological sources

NH^+^_4_ in deep subsurface environments can originate from living biomass which is capable of fixing nitrogen (Holloway and Dahlgren, 2002; Swanner and Templeton, 2011). However, N_2_ fixation is energetically costly, and the question if communities in oligotrophic environments would regularly show nitrogen fixation has been considered by Chivian et al. (2008) and Swanner and Templeton (2011). For the FJQ the indicator genes (*nifH, nifD*) for dinitrogen reduction to ammonium were not detected and therefore the source for the prevailing levels of ammonium in the FJQ remains enigmatic. Admixture of surface and ground waters to the thermal springs of Bad Gastein is variable and depends on their location; the FJQ at an altitude of 1034 m contains the lowest amount of about 6.5% freshwater (Zötl, 1995). Significant production of nitrogen compounds by mineralization of organic matter from leachates or anthropogenic origins is therefore unlikely.

Another possibility is a geological origin of NH^+^_4_ from various types of rocks. Nitrogen-bearing rocks are globally distributed and comprise a potentially large pool of nitrogen that is frequently neglected because of a lack of routine analytical methods for quantification (Holloway and Dahlgren, 2002). There are numerous examples for the presence of NH^+^_4_ in granite, gneiss, mica, biotite, muscovite and others, if only in the ppm range (Honma and Itihara, 1981; Hall, 1999; Holloway and Dahlgren, 2002). Most of these minerals occur in the Gastein valley, where the FJQ is located (Zötl, 1995). The NH^+^_4_ ion is known to exchange readily with K^+^ ions in the minerals and thus is integrated into the crystal lattice (Honma and Itihara, 1981). Hydrothermal alterations of granite and other minerals can release NH^+^_4_ and thereby increase its concentration in deep groundwaters, such as in geysers (Hall, 1999). Although, to our knowledge, the source of ammonium has not been investigated in the alpine subsurface layers and thermal springs, the possibility of a geological origin of ammonium should be taken into consideration. It could provide an explanation for the presence and sustenance of a sizable and viable subterranean microbial community in alpine thermal springs.

In summary, the results suggested the presence of the genetic inventory for a nitrogen cycle in the natural environment of the subsurface thermal mineral spring FJQ. However, only a limited number of sequences of most functional genes for denitrification was recovered, making further work necessary to arrive at a better estimation of the diversity of these genes. The failure of amplification of genes encoding the enzyme nitrogenase might indicate that a geologic source of nitrogen is used in the FJQ, which is supported by the presence of NH^+^_4_ contain minerals in the Gastein valley.

The subsurface environment of the FJQ is a representative habitat with similarities to other geothermal environments. This will allow to address fundamental questions about the composition and activities of the deep biosphere, the possibility of a thermophilic origin of N-transformations, and the implications for the search for life on other planets. To cite Whitman et al. (1998): the absence of detailed knowledge of (the subsurface) prokaryotic diversity represents a major gap in our knowledge of life on earth.

## Author contributions

Friedrich W. Gerbl designed and performed the main research, analyzed data, and created figures; Gerhard W. Weidler analyzed results and contributed phylogenetic data; Wolfgang Wanek performed isotope analysis (IRMS). Angelika Erhardt carried out microcosm and SIP experiments. Friedrich W. Gerbl and Helga Stan-Lotter wrote the manuscript.

### Conflict of interest statement

The authors declare that the research was conducted in the absence of any commercial or financial relationships that could be construed as a potential conflict of interest.
