# Supplementary material for: Thaumarchaeal ammonium oxidation and evidence for a nitrogen cycle in a subsurface radioactive thermal spring in the Austrian Central Alps
Source: Front Microbiol. 2014 May 16;5:225. doi: 10.3389/fmicb.2014.00225 (PMC4032944; doi:10.3389/fmicb.2014.00225)
Supplement: Supplementary file 1 [file DataSheet1.PDF]

Table S1. Phylogenetic relationship of gene sequences encoding nitrogen compound metabolizing enzymes obtained from clone libraries set up with DNA extracted from microcosms. Unc., uncultured.

FG-FJQ clone	N-gene fragment	Closest database sequence	Acc. Nr.	Origin of database sequence	% of Identity
FG-FJQ-amoA-8	archaeal *amoA*	Unc. archaeon clone 16a14	JQ768059	Subsurface thermal spring	100
FG-FJQ-amoA A19-3	archaeal *amoA*	Unc. archaeon clone 16a14	JQ768059	Subsurface thermal spring	99.8
FG-FJQ-amoA-A19-20	archaeal *amoA*	Uncultured archaeon clone B09, amoA gene	JQ768067	Subsurface thermal spring	100
FG-FJQ-amoA-GW-1	archaeal *amoA*	Unc. crenarchaeon partial amoA gene, clone FJQ-OTU13AOA	AM749108	Subsurface thermal spring	98.8
FG-FJQ-amoA-GW-6	archaeal *amoA*	Unc. crenarchaeote partial amoA gene, clone F5	AM260488	Subsurface thermal spring	98.0
FG-FJQ-amoA-WW-1	archaeal *amoA*	Unc. crenarchaeote partial amoA gene, clone F5	AM260488	Subsurface thermal spring	98.8
FG-FJQ-amoA-WW-9	archaeal *amoA*	Unc. crenarchaeon partial amoA gene, clone FJQ-OTU14AOA	AM749109	Subsurface thermal spring	97.7
FG-FJQ-nxrA-2	*nxrA*	nitrate oxidoreductase subunit alpha, *Candidatus* Nitrospira defluvii	YP_003798853	Activated sludge from a municipal WWTP	88.0
FG-FJQ-nxrB-3	*nxrB*	nitrate oxidoreductase subunit beta, *Candidatus* Nitrospira defluvii	YP_003798852	Activated sludge from a municipal WWTP	96.4
FG-FJQ-nxrB-34	*nxrB*	nitrate oxidoreductase subunit beta, *Candidatus* Nitrospira defluvii	YP_003798852	Activated sludge from a municipal WWTP	96.0
FG-FJQ-nxrB-12	*nxrB*	nitrate oxidoreductase subunit beta, *Candidatus* Nitrospira defluvii	YP_003798852	Activated sludge from a municipal WWTP	94.0
FG-FJQ-nxrB-36	*nxrB*	nitrate oxidoreductase subunit beta, *Candidatus* Nitrospira defluvii	YP_003798852	Activated sludge from a municipal WWTP	95.0
FG-FJQ-narG-2	*narG*	nitrate reductase, *Meiothermus silvanus* DSM 9946	CP002042	Hot spring	81.0
FG-FJQ-narG-6	*narG*	Unc. denitrifying bacterium clone V18DBT14 nitrate reductase gene	FJ905011	Coastal aquaculture soil	62.5
FG-FJQ-narG-10	*narG*	Unc. bacterium clone DMG2-248 dissimilatory nitrate reductase gene	EU052897	Lake sediment	87.4
FG-FJQ-nirS-3	*nirS*	Unc. bacterium clone T-H6, nirS gene	HQ428024	Lake sediment	87.4

FG-FJQ-nirS-k2		Unc. bacterium clone ON-S4, nirS gene	JF772712	Rice paddy soil	91.1
FG-FJQ-cnorB-11	*cnorB*	nitric oxide reductase, *Polymorphum gilvum* SL003B-26A1	CP002568	Saline soil	84.1
FG-FJQ-cnorB-12		nitric oxide reductase, *Polymorphum gilvum* SL003B-26A1	CP002568	Saline soil	84.2
FG-FJQ-cnorB-13		Uncultured bacterium clone norB9, cnorB gene	JN559463	Biofilm from a denitrification system	84.1
FG-FJQ-qnorB-8	*qnorB*	Unc. bacterium isolate DGGE gel band BBM4-2 quinol nitric oxide reductase (qnorB) mRNA	FJ866555	Soil	70.7
FG-FJQ-nosZ-3	*nosZ*	nitrous-oxide reductase, *Alicycliphilus denitrificans* K601	CP002657	Anaerobic sewage sludge	82.0
FG-FJQ-nosZ-12		nitrous-oxide reductase, *Alicycliphilus denitrificans* K601	CP002657	Anaerobic sewage sludge	81.5
